# Gene model for the ortholog of *Myc* in *Drosophila eugracilis*

**DOI:** 10.17912/micropub.biology.000912

**Published:** 2024-08-03

**Authors:** Megan E. Lawson, Alexa Hoffman, Isabel G. Wellik, Jeffrey S. Thompson, Joyce Stamm, Chinmay P. Rele

**Affiliations:** 1 The University of Alabama, Tuscaloosa, AL USA; 2 University of Evansville, Evansville, IN USA; 3 Denison University, Granville, OH USA

## Abstract

Gene model for the ortholog of Myc ( *Myc* ) in the *D. eugracilis* Apr. 2013 (BCM-HGSC/Deug_2.0) (DeugGB2) Genome Assembly (GenBank Accession: GCA_000236325.2) of *Drosophila eugracilis* . This ortholog was characterized as part of a developing dataset to study the evolution of the Insulin/insulin-like growth factor signaling pathway (IIS) across the genus *Drosophila* using the Genomics Education Partnership gene annotation protocol for Course-based Undergraduate Research Experiences.

**Figure 1.**
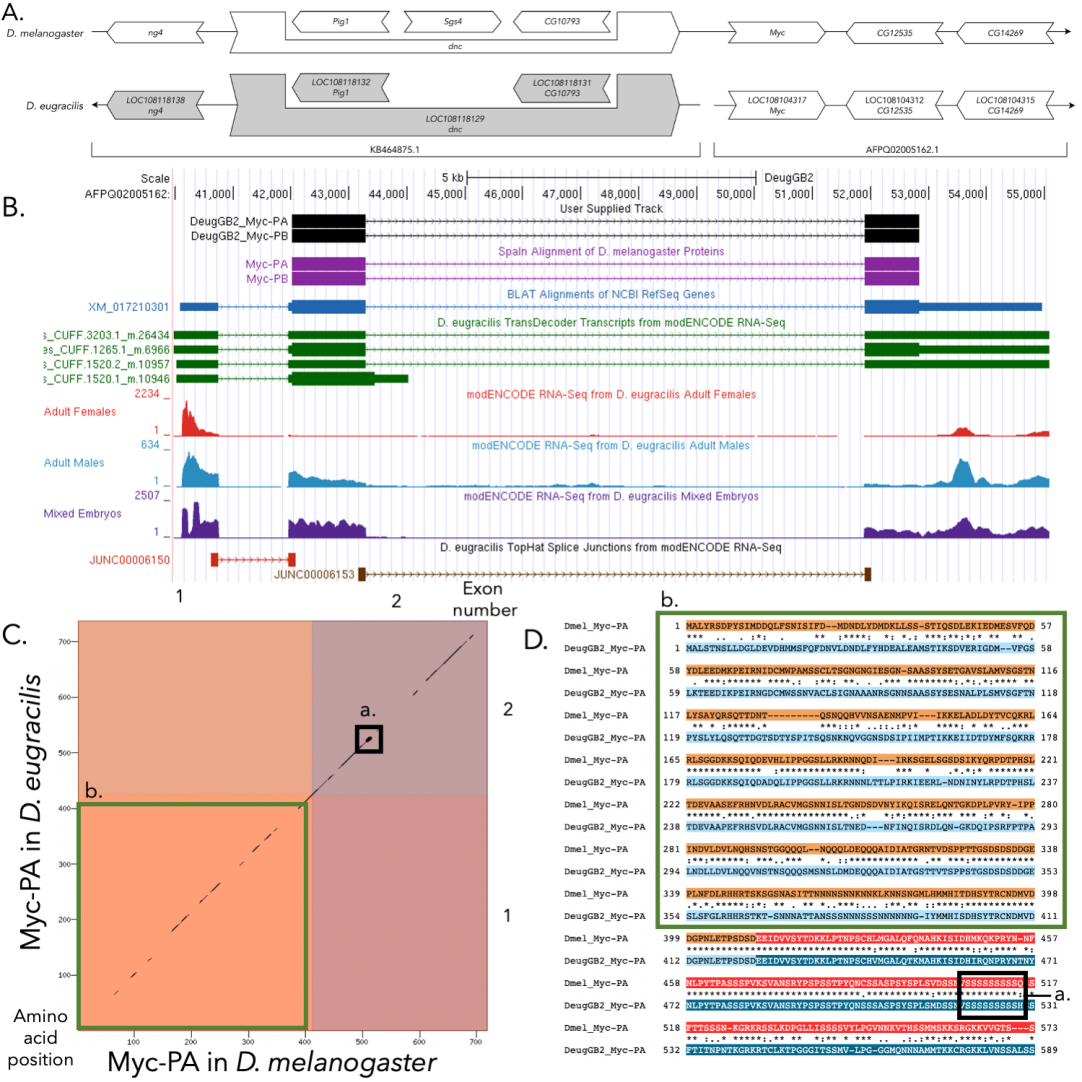
(A) **Synteny of genomic neighborhood of *Myc* in *D. melanogaster* and *D. eugracilis* .** Gene arrows pointing in the same direction as target gene in both *D. eugracilis* and *D. melanogaster* are on the same strand as the target gene; gene arrows pointing in the opposite direction are on the opposite strand. The thin underlying arrows pointing to the right indicate that *Myc* and the downstream genes are on the + strand; the arrow pointing to the left indicates that the upstream genes are on the – strand in *D. eugracilis* . White arrows in *D. eugracilis* indicate the locus ID and the orthology to the corresponding gene in *D. melanogaster,* and gray arrows indicate that the orthologous genes upstream of *Myc* in *D. melanogaster* are found on a different scaffold in *D. eugracilis* than *Myc* . The brackets beneath the local synteny diagram for *D. eugracilis* show which scaffold each gene is found on. The gene names given in the *D. eugracilis* gene arrows indicate the orthologous gene in *D. melanogaster* , while the locus identifiers are specific to *D. eugracilis* . (B) **Gene Model in UCSC Track Hub** (Raney et al. 2014): the gene model in *D. eugracilis* (black), Spaln of D. melanogaster Proteins (purple, alignment of RefSeq proteins from *D. melanogaster* ), BLAT alignments of NCBI RefSeq Genes (blue, alignment of RefSeq genes for *D. eugracilis* ), RNA-seq from female (red), male (blue), and mixed embryos (purple) (alignment of Illumina RNA-seq reads from *D. eugracilis* ), and Transcripts (green) including coding regions predicted by TransDecoder and Splice Junctions Predicted by regtools using D. eugracilis RNA-seq (Chen *et al.* , 2014; PRJNA63469 ). Note that there is no measured expression of the first CDS of *Myc* (Flybase ID: 1_12880_0) in females (Flybase IDs from FB2022_03; Larkin *et al.,* 2021). Splice junctions shown have a minimum read-depth of 702 with 500-999 and >1000 supporting reads in brown and red respectively. The custom gene model (User Supplied Track) is indicated in black with CDSs depicted by boxes and introns by narrow lines (arrows indicate direction of transcription). (C) **Dot Plot of Myc-PA in *D. melanogaster* ( *x* -axis) vs. the orthologous peptide in *D. eugracilis* ( *y* -axis)** . Amino acid number is indicated along the left and bottom; CDS number is indicated along the top and right, and CDSs are also highlighted with alternating colors. The gaps in the dot plot indicate regions with low sequence similarity. The region within the black box (box a) contains a tandem repeat in CDS 2 that is conserved across both *D. melanogaster and D. eugracilis* . The green box (box b) highlights low sequence similarity in CDS one (D) **Idiosyncrasies in the protein alignment.** CDS one, which is boxed in green (box b), has many segments with low amino acid sequence similarity between *D. melanogaster* and *D. eugracilis* . The black box (box a) indicates a tandem repeat in the second CDS.

## Description

**Table:** 

* This article reports a predicted gene model generated by undergraduate work using a structured gene model annotation protocol defined by the Genomics Education Partnership (GEP; thegep.org ) for Course-based Undergraduate Research Experience (CURE). The following information in this box may be repeated in other articles submitted by participants using the same GEP CURE protocol for annotating Drosophila species orthologs of Drosophila melanogaster genes in the insulin signaling pathway. * "In this GEP CURE protocol students use web-based tools to manually annotate genes in non-model *Drosophila* species based on orthology to genes in the well-annotated model organism fruitfly *Drosophila melanogaster* . The GEP uses web-based tools to allow undergraduates to participate in course-based research by generating manual annotations of genes in non-model species (Rele et al., 2023) . Computational-based gene predictions in any organism are often improved by careful manual annotation and curation, allowing for more accurate analyses of gene and genome evolution (Mudge and Harrow 2016; Tello-Ruiz et al., 2019). These models of orthologous genes across species, such as the one presented here, then provide a reliable basis for further evolutionary genomic analyses when made available to the scientific community.” (Myers et al., 2024) . “The particular gene ortholog described here was characterized as part of a developing dataset to study the evolution of the Insulin/insulin-like growth factor signaling pathway (IIS) across the genus *Drosophila* . The Insulin/insulin-like growth factor signaling pathway (IIS) is a highly conserved signaling pathway in animals and is central to mediating organismal responses to nutrients (Hietakangas and Cohen 2009; Grewal 2009) .” (Myers et al., 2024) . “Myc acts downstream of the insulin signaling pathway, with Myc protein accumulating in response to insulin through transcriptional and post-transcriptional mechanisms (Parisi et al., 2011) , resulting in the activation of genes involved in anabolic processes that promote cell growth (Terakawa et al., 2022) . *Myc* encodes a basic helix-loop-helix transcription factor in *Drosophila melanogaster* that is homologous to vertebrate *Myc* proto-oncogenes (Gallant et al., 1996) . In *Drosophila melanogaster* , Myc transcriptionally regulates a wide range of genes, including those that influence cell growth and metabolism (Teleman et al., 2008; Gallant 2013) .” (Myers et al., 2024) . “ *D. eugracilis* (NCBI:txid29029) is part of the *melanogaste* r species group within the subgenus *Sophophora* of the genus *Drosophila* (Pélandakis et al., 1993) . It was first described as *Tanygastrella gracilis* by Duda (1924) and revised to *Drosophila eugracilis* by Bock and Wheeler (1972). *D. eugracilis* is found in humid tropical and subtropical forests across southeast Asia (https://www.taxodros.uzh.ch, accessed 1 Feb 2023).” (Morgan et al., 2022) .

The model presented here is the ortholog of *Myc* in the Apr. 2013 (BCM-HGSC/Deug_2.0) assembly of *D. eugracilis* ( GCA_000236325.2 *)* and corresponds to the *Gnomon Peptide ID ( XP_017065790.1 )* predicted model in *D. eugracilis ( LOC108104317 ).* This gene model is based on RNA-seq data from *D. eugracilis* (Chen et al., 2014; PRJNA63469 ) and the *Myc* (Drosophila 12 Genomes Consortium et al *.* , 2007; *GCA_000001215.4* ) in *D. melanogaster* from FB2023_03 ( GCA_000001215.4 ; Larkin et al., 2021; Gramates et al., 2022).

Gene and species details can be found in the description above.

***Synteny***

*Myc* occurs on chromosome X in *D. melanogaster* and is flanked by *ng4* and *dnc* upstream and *CG12535* and *CG14269* downstream *.* The upstream *dnc* gene in *D. melanogaster* nests *Pig1, Sgs4,* and *CG10793 .* We determined that the putative ortholog of *Myc* is found on the AFPQ02005162.1 scaffold in *D. eugracilis* (GB2 assembly GCA_000236325.2 ) with LOC108104317 ( XP_017065790.1 ) (via *tblastn* search with an e-value of 0.0 and percent identity of 68.18%). It is flanked downstream by LOC108104312 ( XP_017065785.1 ) and LOC108104315 ( XP_017065789.1 ), which correspond to *CG12535* and *CG14269 in D. melanogaster* with e-values of 7e-72 and 6e-110 respectively, and percent identities of 55.83% and 79.47% respectively, as determined by *blastp* ( Figure 1A, Altschul et al ., 1990). *Myc* is the first gene on the AFPQ02005162.1 scaffold in *D. eugracilis* , so there are no upstream genes to analyze for local synteny. However, *blastp* results indicated that the orthologs of genes upstream of *Myc* in *D. melanogaster* are located on scaffold KB464875.1 in *D. eugracilis* with LOC108118138 ( XP_017086187.1 ) *,* LOC108118129 ( XP_041674349.1 ) *,* LOC108118132 ( XP_017086181.1 ) *,* and LOC108118131 ( XP_017086180.1 ), which correspond to *ng4, dnc, Pig1,* and *CG10793* with e-values of 9e-22, 0.0, 2e-77, and 0.0, respectively, and percent identities of 82.76%, 95.42%, 58.33%, and 87.81% respectively, as determined by *blastp* ( Figure 1A, Altschul et al ., 1990). Local synteny was conserved within this part of the neighborhood in *D. eugracilis* as well, with the exception of *Sgs4* , for which an ortholog in *D. eugracilis* could not be located. We believe this is the correct ortholog assignment for *Myc* in *D. eugracilis* because all of the *BLAST* hits had very low e-values and were the best *BLAST* result by a wide margin, and because local synteny is well-conserved throughout the genomic neighborhood.

***Protein Model***

*Myc* in *D. eugracilis* has one unique protein coding isoform encoded by mRNAs Myc-RA and Myc-RB, which differ in their UTRs ( Figure 1B ). Myc-PA/Myc-PB contain two protein coding CDSs. This is the same relative to the ortholog in *D. melanogaster.* The sequence of Myc in *D. eugracilis* has 67.0% identity with *Myc* in *D. melanogaster* as determined by *blastp* ( Figure 1C ). This is more divergence between the sequence than one would expect, considering how closely related *D. melanogaster* and *D. eugracilis* are. The coordinates of the curated gene models can be found in NCBI at GenBank/BankIt using the accessions BK063012 and BK063013 . These data are also available in Extended Data files below, which are archived in CaltechData.

***Special characteristics of the protein model***

**Tandem repeat in CDS two**

There is a tandem repeat present in CDS two in *Myc* , which is shown in figures C and D in black box a. Specifically, the repeat is made up of nine uninterrupted Serine amino acids present in the protein sequence.

**Low sequence similarity in CDS one**

There are many regions in CDS one of *Myc* that have very low conservation of their amino acid sequences between the two species. This is pictured in the green boxes (box b) in figures C and D.

## Methods

Detailed methods including algorithms, database versions, and citations for the complete annotation process can be found in Rele et al. (2023). Briefly, students use the GEP instance of the UCSC Genome Browser v.435 ( https://gander.wustl.edu ; Kent WJ et al., 2002; Navarro Gonzalez et al., 2021) to examine the genomic neighborhood of their reference IIS gene in the *D. melanogaster* genome assembly (Aug. 2014; BDGP Release 6 + ISO1 MT/dm6). Students then retrieve the protein sequence for the *D. melanogaster* target gene for a given isoform and run it using *tblastn* against their target *Drosophila* species genome assembly ( *D. eugracilis* ( GCA_000236325.2 *)* ) on the NCBI BLAST server ( https://blast.ncbi.nlm.nih.gov/Blast.cgi , Altschul et al., 1990) to identify potential orthologs. To validate the potential ortholog, students compare the local genomic neighborhood of their potential ortholog with the genomic neighborhood of their reference gene in *D. melanogaster* . This local synteny analysis includes at minimum the two upstream and downstream genes relative to their putative ortholog. They also explore other sets of genomic evidence using multiple alignment tracks in the Genome Browser, including BLAT alignments of RefSeq Genes, Spaln alignment of D. melanogaster proteins, multiple gene prediction tracks (e.g., GeMoMa, Geneid, Augustus), and modENCODE RNA-Seq from the target species. Genomic structure information (e.g., CDSs, CDS number and boundaries, number of isoforms) for the *D. melanogaster* reference gene is retrieved through the Gene Record Finder ( https://gander.wustl.edu/~wilson/dmelgenerecord/index.html ; Rele et al *.,* 2023). Approximate splice sites within the target gene are determined using *tblastn* using the CDSs from the *D. melanogaste* r reference gene. Coordinates of CDSs are then refined by examining aligned modENCODE RNA-Seq data, and by applying paradigms of molecular biology such as identifying canonical splice site sequences and ensuring the maintenance of an open reading frame across hypothesized splice sites. Students then confirm the biological validity of their target gene model using the Gene Model Checker ( https://gander.wustl.edu/~wilson/dmelgenerecord/index.html ; Rele et al., 2023), which compares the structure and translated sequence from their hypothesized target gene model against the *D. melanogaster* reference gene model. At least two independent models for this gene were generated by students under mentorship of their faculty course instructors. These models were then reconciled by a third independent researcher mentored by the project leaders to produce the final model presented here. Note: comparison of 5' and 3' UTR sequence information is not included in this GEP CURE protocol.

## Extended Data

Description: GFF, FASTA, and PEP of the model. Resource Type: Model. DOI: 10.22002/wtccf-p2596

Description: response to editorial pre-review. Resource Type: Text. DOI: 10.22002/c7079-pkh57
